# Evaluating Amazon's Mechanical Turk as a Tool for Experimental Behavioral Research

**DOI:** 10.1371/journal.pone.0057410

**Published:** 2013-03-13

**Authors:** Matthew J. C. Crump, John V. McDonnell, Todd M. Gureckis

**Affiliations:** 1 Department of Psychology, Brooklyn College of CUNY, Brooklyn, New York, United States of America; 2 Department of Psychology, New York University, New York, New York, United States of America; University College London, United Kingdom

## Abstract

Amazon Mechanical Turk (AMT) is an online crowdsourcing service where anonymous online workers complete web-based tasks for small sums of money. The service has attracted attention from experimental psychologists interested in gathering human subject data more efficiently. However, relative to traditional laboratory studies, many aspects of the testing environment are not under the experimenter's control. In this paper, we attempt to empirically evaluate the fidelity of the AMT system for use in cognitive behavioral experiments. These types of experiment differ from simple surveys in that they require multiple trials, sustained attention from participants, comprehension of complex instructions, and millisecond accuracy for response recording and stimulus presentation. We replicate a diverse body of tasks from experimental psychology including the Stroop, Switching, Flanker, Simon, Posner Cuing, attentional blink, subliminal priming, and category learning tasks using participants recruited using AMT. While most of replications were qualitatively successful and validated the approach of collecting data anonymously online using a web-browser, others revealed disparity between laboratory results and online results. A number of important lessons were encountered in the process of conducting these replications that should be of value to other researchers.

## Introduction

One challenging aspect of experimental psychology research is the constant struggle for data. Typically, researchers depend on university undergraduates who participate in studies in exchange for experience, course credit, or money. Research progress depends on the ebb and flow of the semester. As a result, it can take weeks, months, or even years to conduct a large behavioral study. This issue is even more salient for researchers at smaller universities.

One appealing solution is to collect behavioral data over the Internet. In theory, online experimentation would allow researchers to access to a large and diverse pool of potential subjects worldwide, using automated replicable techniques free of unintended experimenter effects. However, the main obstacle to conducting Internet-based research is finding people who are willing to participate and compensating them.

Recently, a number of online crowdsourcing services have been developed which connect individuals willing to perform online tasks with other individuals willing to pay for work to be done. Perhaps the most popular system is Amazon's Mechanical Turk (AMT). AMT is useful for behavioral researchers because it handles recruitment and payment in a fairly automatic way. Most importantly, there are a large number of people who use AMT making it a great way to advertise and distribute studies (over 100,000 active users in 2007 [1]).

There are a number of recent summaries about using AMT for research [2]. In addition, the service has been validated as a tool for conducting survey research [3], [4], one-shot decision-making research [5], [6], collective behavior experiments [7], [8], for norming stimuli, and conducting behavioral linguistics experiments [9], [10].

However, less is known about the viability of conducting behavioral experiments typical of those used in cognitive science and cognitive psychology. Such studies are unique in that they typically involve multi-trial designs, sustained attention on the part of participants, millisecond timing for response recording and stimulus presentation, and relatively complex instructions. These features present two key challenges for online data collection. First, there are technical challenges in programming web-based experiment protocols and then ensuring the browser systems of the participant and experimenter support the same features. Second, experiments where memory and timing are important are likely more sensitive to incidental aspects of the testing environment that are difficult to control online (e.g., presence of distractions, problems with display, pausing for long periods in the middle of the task, and misreading or misunderstanding of instructions).

The aim of the present paper is to validate AMT as a tool for behavioral cognitive research, with a specific focus on complex multi-trial designs. We focus on AMT simply because it is the most popular system currently available and the one most researchers would likely consider. If the data obtained from AMT can replicate classic findings in the field with reasonable fidelity, it will validate the potential of the service for use in cognitive behavioral research. In this sense our study joins a number of recent articles exploring the relationship between data collected online and in the lab [11], [3]. However, unlike existing work, in the present study we focus on *qualitative* replication of theoretically significant findings rather than on comparisons of the performance variance or mean performance of lab and online participants (e.g., [11]). The standard of qualitative replication (i.e., the ability to detect reliable differences as the result of an established and widely accepted experimental manipulation) is likely the one of most interest to researchers and has the greatest importance for the field.

In the present study we attempted to replicate influential findings from several classic cognitive phenomena from the attention, performance, and learning literatures. The replication studies were chosen to represent and validate a broad range of multi-trial designs that require millisecond control over response collection and stimulus presentation, as well as designs that require complex decision making and where task instructions are critically important. To foreshadow, our results suggest that data collected online using AMT closely resemble data collected in the lab under more controlled situations. However, for certain types of experiments the alignment between laboratory results and online results showed greater disparity. In the conclusion of the paper we suggest some general lessons we obtained which may help inform other researchers considering using online data in their work.

### AMT Basics

There are a number of in-depth overviews of using AMT to conduct behavioral experiments [4], [2], [6]. In brief, the service allows individuals (known as *requesters*) to post *human intelligence tasks* (HITs) that other individuals (known as *workers*) can complete for small sums of money. Each HIT is a small unit of work that is typically completed in the worker's web browser. HITs can be composed of assignments which allow multiple workers to complete the same HIT. Common HITs include providing keywords for the objects in an image or giving feedback about a website. These tasks typically require some form of human intelligence, but can be completed by almost anyone in a few minutes. In the present case, we consider a HIT to be a request to participate in an entire cognitive experiment.

Amazon provides web-based, point-and-click tools for creating several different kinds of HITs; however none of these tools are suitable for the control and flexibility necessary for conducting complex behavioral experiments. As an alternative, Amazon provides a way for tasks to be completed on an external webserver. As a result, requesters (in this case, psychologists) need only program and host an external website that is capable of running the desired experiment. Any task that can be programmed using standard web browser technology (e.g., HTML with JavaScript or Flash) can be used on AMT. Once the external website HIT has been designed, it must be set up to interface with Amazon's service so that workers who accept and complete the task can be paid (see [2] for details).

Once a HIT is posted to the service it will be available for workers to complete. Restrictions can be set to limit HIT completions to workers with unique *worker ID*s (a unique number assigned to each worker when they sign up), or to workers of a certain age or from a certain region. Workers that qualify for the HIT can view a short task description along with the pay rate, and choose whether or not to accept the task. A worker clicks an accept button when they decide to complete the HIT and a submit button when they have completed the HIT. At any time, the worker can choose to stop and return the HIT to the requester. This allows another worker to complete the HIT instead. The requester also has the option to reject payment for unsatisfactory work. Payment is usually handled through a credit card and processed through Amazon's payments system.

### Potential Advantages and Disadvantages of Collecting Data Online

There are many reasons researchers may be enthusiastic about collecting behavioral data online [12] using services such as AMT. First, data can be collected more quickly than in the lab. Second, since the experimenter never directly meets or interacts with the anonymous participants, it minimizes the chance that the experimenter can influence the results. In addition, the code for such experiments can easily be shared online to other researchers to facilitate replication with a more or less identical population sample. Finally, studies have shown that AMT workers are generally more diverse than undergraduate college students and are instead representative of the general demographics of the Internet-using population [13], [2], [7], [14], [8].

However, there are a number of limitations facing researchers running experiments on AMT. First, workers are kept completely anonymous as part of Amazon's terms of service making it difficult to verify demographic information (and people may not truthfully answer certain questions on demographic surveys). Second, the computer systems that workers use to complete HITs should be assumed to vary widely and the error in measuring reaction time data and ensuring precise timing of stimulus displays is unknown. Third, there is a complete lack of environmental control. For example, workers could be simultaneously watching TV or cooking breakfast while performing the task. This could have negative consequences, particularly on tasks where subject must learn or memorize something from one trial to the next. Finally, although the service is assumed to involve human workers there is a possibility of non-human workers (i.e., bots) that may try to subvert the design in order to obtain payment. Together, these concerns could limit the usefulness of service for conducting behavioral experiments.

One way to address these issues is technical (e.g., introducing screening questions that cannot be reasonably answered by bots, requiring trials to be completed quickly and accurately, etc…). However, an important validation of the system may be obtained through empirical analysis. If the system can be used to replicate well-known and widely replicated laboratory findings from cognitive psychology, researchers can pursue novel scientific questions with greater confidence (Tthis is the standard that we believe most researchers would intuitively use to judge the usefulness of such systems.) This is the approach we have taken in the experiments that follow.

### Empirical Validation through Replication

The purpose of the present experiments is to validate AMT as a tool for running multi-trial designs that are common in behavioral cognitive research. The experiments were chosen first to give a broad, representative sample of the kinds of tasks that are typically used in the field, and second, to satisfy three main validation criteria that would be important to many researchers. Three series of experiments were run: The first to validate multi-trial designs requiring millisecond control over response collection; the second to validate multi-trial designs requiring millisecond control over stimulus presentation; and the third to validate other aspects of multi-trial designs, with a focus on experiments where instructional manipulations are important. The experiments were all conducted on AMT in a joint effort across the labs of the first and last author. As such, there are minor differences in the general experimental protocols (e.g., method of consent, subject payment) employed in the coding of the web-based experiments. The experiments in the first and second series were coded together by the first author, and the experiments in the third series were coded together by the second and last author. The series of experiments are reported in turn below.

### Ethics Statement

The experiments reported in Section 1 and Section 2 were approved and in compliance with the Brooklyn College Institutional Review Board. The experiments reported in Section 3 were approved and in compliance with the New York University Institutional Review Board.

## Section 1: Reaction Time Experiments

Many cognitive tasks require millisecond timing for response collection. Reaction time measurements are inherently noisy, people are sometimes fast and sometimes slow, and researchers commonly employ multi-trial designs to reduce measurement error. Measurement error is also reduced in lab-based research using software and hardware that can guarantee millisecond precision. This guarantee is likely impossible to duplicate inside a web browser.

JavaScript running in modern web browsers has millisecond timing capability and this allows for some control over stimulus presentation rates and response recording [15]. However, even though JavaScript records millisecond timestamps, timing variability for the sampling rate on any given subject's computer is unknown. Keyboards have different sampling rates monitors have different refresh rates, and different web browsers running on different computers have highly variable presentation lags. Nevertheless, these timing errors may be relatively small and almost certainly random across subjects [16]. Typically, participants' response times are considerably more variable than their computer systems' timing errors. Indeed, prior work using Flash-based programming to collect online data have successfully replicated simple binary-choice RT effects [17] and task-switching effects [18].

Continuing in this vein to validate AMT as a tool to conduct reaction time research, several classic reaction time effects were replicated. The replications included Stroop, task-switching, Eriksen flanker, Simon, and Posner cuing tasks. The Posner cuing task is included in Section 2 as it also requires precise control over stimulus presentation.

Unless otherwise noted, the experiments involve short 5 min tasks involving approximately 100 trials. Each experiment was loaded as a single HIT to Amazon Turk with 60 available assignments. The experiments reported here have varying numbers of participants, as it is common for some proportion of the requested HITs to be returned incomplete. If a specific number of participants is required it would be easy to request enough HITs to ensure the required number of successful completions. Equal numbers of subjects per condition were not obtained to give the reader a better sense of subject attrition and HIT completion rate. For all experiments in section one, participants electronically signed consent forms. The Brooklyn College Institutional Review Board approved the study designs.

## Experiment 1: Stroop

The Stroop task is a classic multi-trial procedure involving ink-color identification of congruent (the word *blue* in blue) or incongruent (*blue* in red) word-color pairs [19], [20]. There are many variants of the response mode in the identification task ranging from vocal naming, pressing arbitrary keys for assigned colors, and typing out the required response. All these variants produce faster response times for congruent than incongruent items, and this difference is termed the *Stroop effect*. The present replication created Stroop stimuli using the colors red, green, blue, and yellow. The design employed a typing identification response, which is known to produce large Stroop effects [21]. An equal proportion of congruent and incongruent trials were presented over the course of 96 trials.

### Methods

#### Participants

One HIT with 60 assignments was loaded onto AMT. Forty unique workers completed all 96 trials. Prior to the experiment workers verified their typing ability by copying a sentence as quickly and accurately as possible. If the sentence was typed faster than 40 words/min, the worker continued to the main task. Mean words/min was 53. Demographic information was not collected and workers remained completely anonymous. Workers were paid $0.10 to complete the task, which lasted approximately 5 min.

#### Apparatus, Stimuli & Design

The experiment was presented to workers as an HTML webpage with task flow controlled by JavaScript code running locally in each worker's web browser.

Stroop trials were constructed from pairing the colors red, green, blue, and yellow with their respective English words, resulting in four possible congruent and 12 incongruent items. The words were presented in 50-pt font in the center of the webpage. The background color of the page was black. There were a total of 96 trials with 48 congruent and 48 incongruent items.

#### Procedure

Workers on AMT found the experiment by browsing for HITs on Amazon's website. When viewing the experiment “ad” workers were presented with a webpage containing task instructions and informed consent. Workers could view example trials so they could decide whether the task was of interest before accepting the HIT by pressing a button. Next, they viewed a verification screen and were asked to type the sentence as quickly and accurately as possible (described above).

The main task consisted of a resizable webpage with a black background. The top left corner contained a small button with the label, “submit when all trials completed.” Pressing this button sent the collected data to Amazon and confirmed with Amazon that the worker completed the task. Below the submit button was an instruction button that would display task instructions if a reminder was necessary. Below this was a trial counter showing the current trial number and number left to be completed. Workers could press the submit button at any time during the experiment.

Each trial began with a central fixation cross for 500 ms and a blank interval for 500 ms followed immediately by a Stroop item that remained on screen until responses were submitted. Subjects typed the name of the ink-color in full then pressed the spacebar to submit their response. The backspace key was disabled and subjects were prevented from correcting their responses. Typed responses were echoed on screen as feedback directly below the target stimulus in white 50-pt font. Spacebar presses cleared the Stroop item and typed response from the screen and triggered presentation of accuracy feedback in the form of the words “correct” or “incorrect”, which were presented above the target stimulus location in white 50-pt font. This feedback was presented on screen for 500 ms and was removed at the beginning of the next trial, which was automatically triggered.

### Results and Discussion

**Figure 1:**
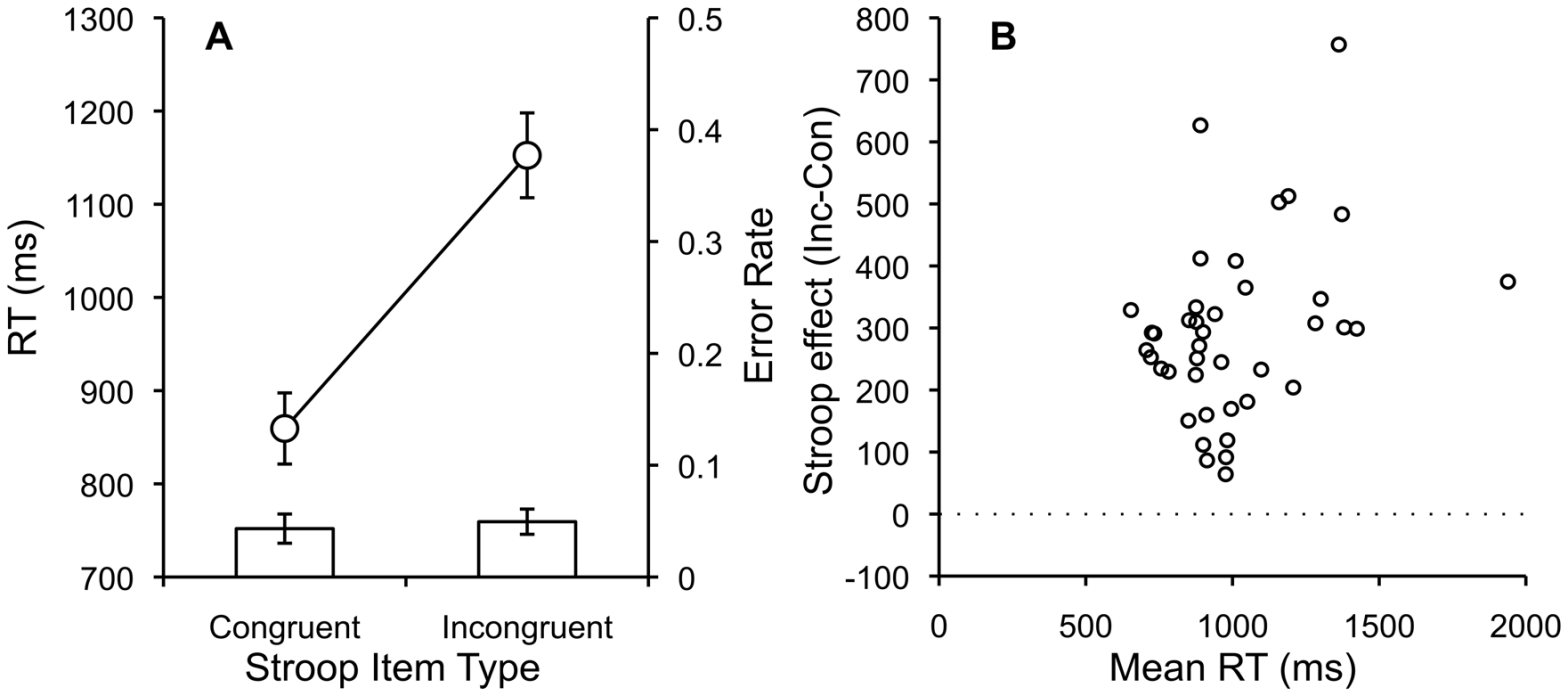
Congruent and Incongruent RTs, Error Rates and Individual Stroop Scores by Mean RT. A. Mean RTs and error rates for congruent and incongruent Stroop items with standard error bars. B. Individual subject Stroop difference scores (incongruent-congruent) plotted as a function of individual subject mean RTs.

Reaction times (RT) were defined as the time between the onset of the Stroop stimulus and the first keystroke to type the color name. Only correct trials where subjects typed the entire color name correctly were analyzed. RTs for each subject in each condition were submitted to an outlier analysis [22], which removed 3% of the observations. Mean RTs and error rates for each subject in each condition were submitted to separate one-way repeated measures ANOVAs with congruency (congruent vs. incongruent) as the single factor. Figure 1a shows mean RTs and error rates for each condition, and Figure 1b shows individual subject variability with individual Stroop difference scores plotted against mean RTs.

RTs were significantly faster for congruent (859 ms) than incongruent (1,152 ms) trials, F(1,39) = 179.80, MSE = 11461.39, p<.001, η^2^_p_ = .82, showing a large (293 ms) Stroop effect. Error rates were low overall and the smaller error rates for congruent (.045) than incongruent items (.059) was marginally significant, F(1,39) = 3.51, MSE = 0.0013, p<.068, η^2^_p_ = .08.

These results replicate the classic Stroop effect. Observed RT values were consistent with Logan & Zbrodoff [21], who reported 809 ms for congruent and 1,023 ms for incongruent items. Error rates were low, showing that participants were capable of understanding and performing the task according to the instructions. This provides a first demonstration that classic attention and performance effects can be obtained using Amazon Turk.

## Experiment 2: Task-Switching Costs

Task performance is generally faster and more accurate when the same task is repeated over trials and slower and more error prone when task demands alternate over trials. This effect is termed the task-switch cost [23], [24], [25]. We tested for switching costs on AMT using a standard procedure. Subjects were given one of two task cues along with a target digit (1–4, or 6–9): The task cue “ODD/EVEN” instructed subjects to judge whether the target was odd or even, while the task cue “SMALL/BIG” instructed subjects to judge whether the target was smaller or bigger than five. The two tasks alternated randomly throughout the experiment. The key dependent measure was RT as a function of if the task switched or repeated from the previous trial.

### Methods

#### Participants

One HIT with 60 assignments was submitted to AMT.

Fifty-five unique workers completed all 96 trials. Demographic information was not collected and workers remained completely anonymous. Workers were paid $0.10 to complete the task, which lasted approximately 5 min.

#### Apparatus, Stimuli & Design

As in Experiment 1, the experiment was presented to workers as an HTML webpage with task flow controlled by JavaScript code running locally in each worker's web browser.

Target items were the integer numbers ‘1’ through ‘9’ with the exception of ‘5’. Targets and task cues were presented in white, 50-pt font, on a black background. The odd and even responses were given using the ‘A’ and ‘S’ keys, respectively. The small and big responses were given using the ‘K’ and ‘L’ keys, respectively. Feedback for correct and incorrect trials was presented in white using 50 pt font. There were a total of 96 trials, with 50% odd/even and small/big task cues. Which task was presented on a given trial was randomly determined for each subject.

#### Procedure

The same general web-based procedure used in Experiment 1 was employed here. Each trial began with a fixation point displayed for 500 ms, followed immediately by a task-cue and target stimulus that remained on the screen until the response. Cues and targets were presented centrally, with the cue presented directly above the target. Subjects were instructed to make their responses as quickly and accurately as possible. At the time of the response, cues and targets were immediately replaced with feedback indicating whether the response was correct or incorrect. The next trial was triggered automatically with a delay of 500 ms.

### Results & Discussion

**Figure 2:**
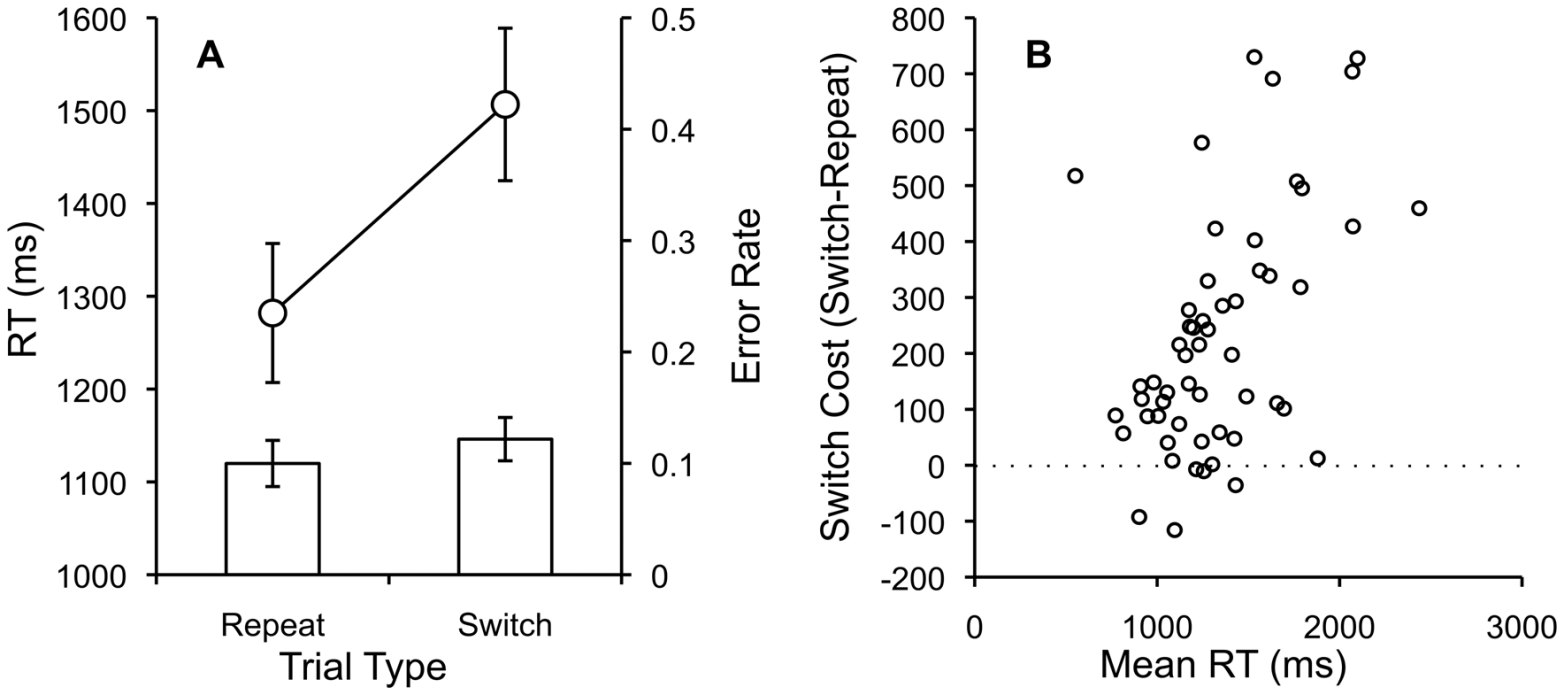
Repeat and Switch RTs, Error Rates and Individual Switch costs by mean RT. A. Mean RTs and error rates for task repeat and switch trials with standard error bars. B. Individual subject switch costs (switch-repeat) plotted as a function of individual subject mean RTs.

The same outlier analyses applied in Experiment 1 resulted in the removal of 3% of the data from each condition. Mean RTs and error rates for each subject in each condition were submitted to separate one-way repeated measures ANOVAs with switching (repeat vs. switch) as the single factor. Figure 2a shows mean RTs and error rates for each condition, Figure 2b shows individual subject switch costs plotted as a function of mean RT.

RTs were significantly faster for repeat (1282 ms) than switch (1507 ms) trials, F(1,54) = 61.56, MSE = 22556.99, p<.001, η^2^_p_ = .53, showing large switch-costs (225 ms). Error rates were low overall and significantly lower for repeat (.1) than switch trials (.12), F(1,54) = 9.77, MSE = .0013, p<.003, η^2^_p_ = .15.

Task-switch costs were observed providing another demonstration that AMT can replicate classic RT effects in the attention domain. During the review process we learned that this is not the first report of using the web to measure task-switch costs. Reimers & Maylor [18] conducted a large (n = 5,271) online study (but not on AMT) using a Flash-based website to measure task-switching performance across the ages 10–66. Our mean switch costs, RTs, and error rates fall within their reported ranges, showing qualitative replication of the web-based approach across different online recruiting approaches and experimental apparatus.

## Experiment 3: Flanker

The Flanker task [26], [27] measures participants' spatial attention in a task requiring them to select relevant from irrelevant information. Flanker stimuli are typically rows of letters. Subjects are instructed to identify the centrally presented target as quickly and accurately as possible. Compatible (e.g., hhhhh) or incompatible (e.g., ffhff) distractors flank a central target. Correct responses thus require the subject to ignore the distractors and respond only based on the target. Typically, the flanking letters are among the possible targets, and RTs are faster for compatible than incompatible trials. This is often taken to imply that the distractors are being processed to some degree even when they should be ignored.

### Methods

#### Participants

One HIT with 60 assignments was submitted to AMT. Fifty-two unique workers completed all 96 trials. Demographic information was not collected and workers remained completely anonymous. Workers were paid $0.10 to complete the task which lasted approximately 5 min.

#### Apparatus, Stimuli & Design

The experiment was again presented to workers as an HTML webpage with task flow controlled by JavaScript code running locally in each worker's web browser.

Flanker items were constructed from the lowercase letters ‘f’ and ‘h’. There were two compatible items (‘fffff’, ‘hhhhh’) and two incompatible trials (‘ffhff’, ‘hhfhh’). The keyboard responses ‘F’ and ‘H’ were used to identify the targets. The display parameters were the same as the Stroop experiment. Flanker stimuli were presented in white on a black webpage background in 50-pt font. Feedback for correct and incorrect trials was presented in white using 50-pt font. There were a total of 100 trials, with 50% compatible and incompatible items. On each trial a random procedure was used to determine which of the four items was presented, and the random trial sequence was different for each subject.

#### Procedure

The same general web-based procedure used in Experiments 1 and 2 was employed here. Each trial began with a fixation point displayed for 500 ms, followed immediately by a Flanker stimulus that remained onscreen until the response. Subjects made their response by pressing the ‘F’ or ‘H’ key as quickly and accurately as possible. This button press immediately replaced the Flanker stimulus with feedback indicating whether the response was correct or incorrect. The next trial was triggered automatically with a delay of 500 ms.

### Results & Discussion

**Figure 3:**
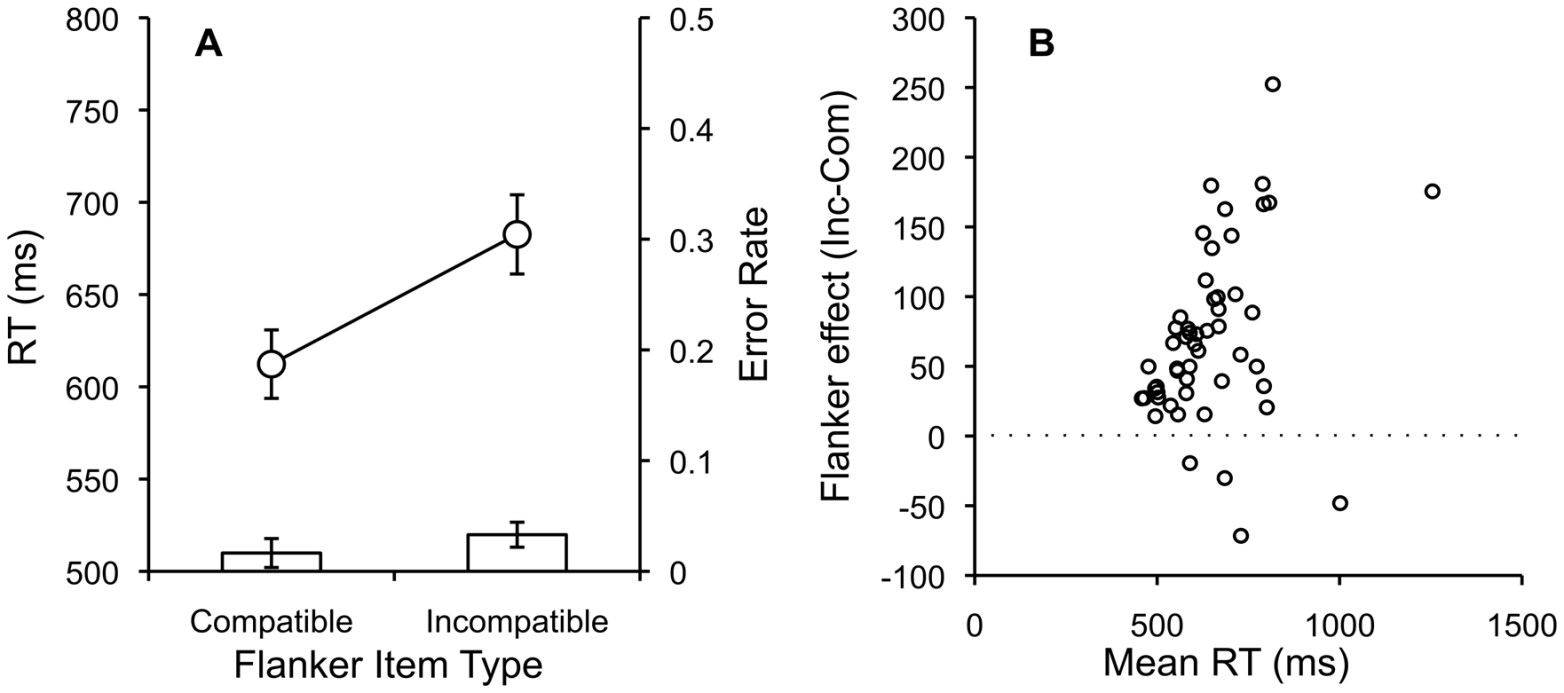
Compatible and Incompatible RTs, Error Rates and Individual Flanker Scores by Mean RT. A. Mean RTs and error rates for compatible and incompatible flanker items with standard error bars. B. Individual subject flanker scores (incompatible-compatible) plotted as a function of individual subject mean RTs.

The same outlier analyses applied in Experiments 1 and 2 resulted in removal 3% of the data from each condition. Mean RTs and error rates for each subject in each condition were submitted to separate one-way repeated measures ANOVAs with compatibility (compatible vs. incompatible) as the single factor. Figure 3A shows mean RTs and error rates for each condition, and Figure 3B shows individual subject flanker difference scores plotted as a function of mean RT.

RTs were significantly faster for compatible (612 ms) than incompatible (682 ms) trials, F(1,51) = 65.78, MSE = 1954.10, p<.001, η^2^_p_ = .56, showing a typical Flanker effect. Error rates were low overall and significantly lower for compatible (.016) than incompatible (.033) trials, F(1,51) = 10.33, MSE = .00069, p<.003 η^2^_p_ = .17.

Overall, the Flanker effect was replicated. RTs and error rates appear within in the range reported in laboratory studies. For example, using a related procedure Wendt & Kiesel [28] found similar RTs for compatible (604 ms) and incompatible trials (647 ms). Across experiments, the Stroop, task-switching and Flanker effects reported involved fairly large RT differences. Clearly these very strong effects can survive whatever noise is inherent to collecting data through AMT. However, a natural question is whether smaller RT differences can also be detected.

## Experiment 4: Simon

The Simon task [29], [30] measures spatial compatibility effects in choice-reaction time. In a typical design targets are presented in one of two visual locations, and responses are made with a left or right button press. For example, red targets would be identified with a left button and green targets with a right button. RTs are usually faster for visual target presented in a location that is spatially compatible with the response (e.g., when the red item occurs on the left side of the screen) than when the target is placed in a spatially incompatible location (e.g., the red item occurs on the right side of the screen). Simon effects are typically much smaller in size than Stroop, Task-switching, and Flanker effects. Thus, replicating these effects would further validate AMT as tool for detecting small RT differences.

### Methods

#### Participants

One HIT with 60 assignments was submitted to AMT. Fifty-eight unique workers completed all 96 trials. Demographic information was not collected and workers remained completely anonymous. Workers were paid $0.10 to complete the task, which lasted approximately 5 min.

#### Apparatus, Stimuli & Design

The experiment was presented to workers as an HTML webpage with task flow controlled by JavaScript code running locally in each worker's web browser.

The display consisted of three placeholder squares 150 px on each side, placed on the left, center, and right side of the screen. Placeholders were separated by 170 px. Each placeholder square was a black-filled square with a white border presented on a black background. Target items were red and green squares 100 px per side. Targets could appear in the right or left locations. The response for the red square was the ‘S’ key located on the left side of the keyboard, and the response for the green square was the ‘K’ key located on the right side of the keyboard. There were a total of 100 trials, with 50% spatially compatible and incompatible trials. On each trial the location of the target and the color was randomly determined, and the random sequence of trials was unique for each participant.

#### Procedure

The same general web-based procedure used in Experiment 1–3 was employed here. Each trial began with a fixation point displayed for 500 ms, followed immediately by a target square that remained onscreen until the response. Subjects were instructed to make their responses as quickly and accurately as possible. Responses immediately removed the placeholders and target from the screen and were followed immediately by feedback indicating whether the response was correct or incorrect. The next trial was triggered automatically with a delay of 500 ms.

### Results & Discussion

**Figure 4:**
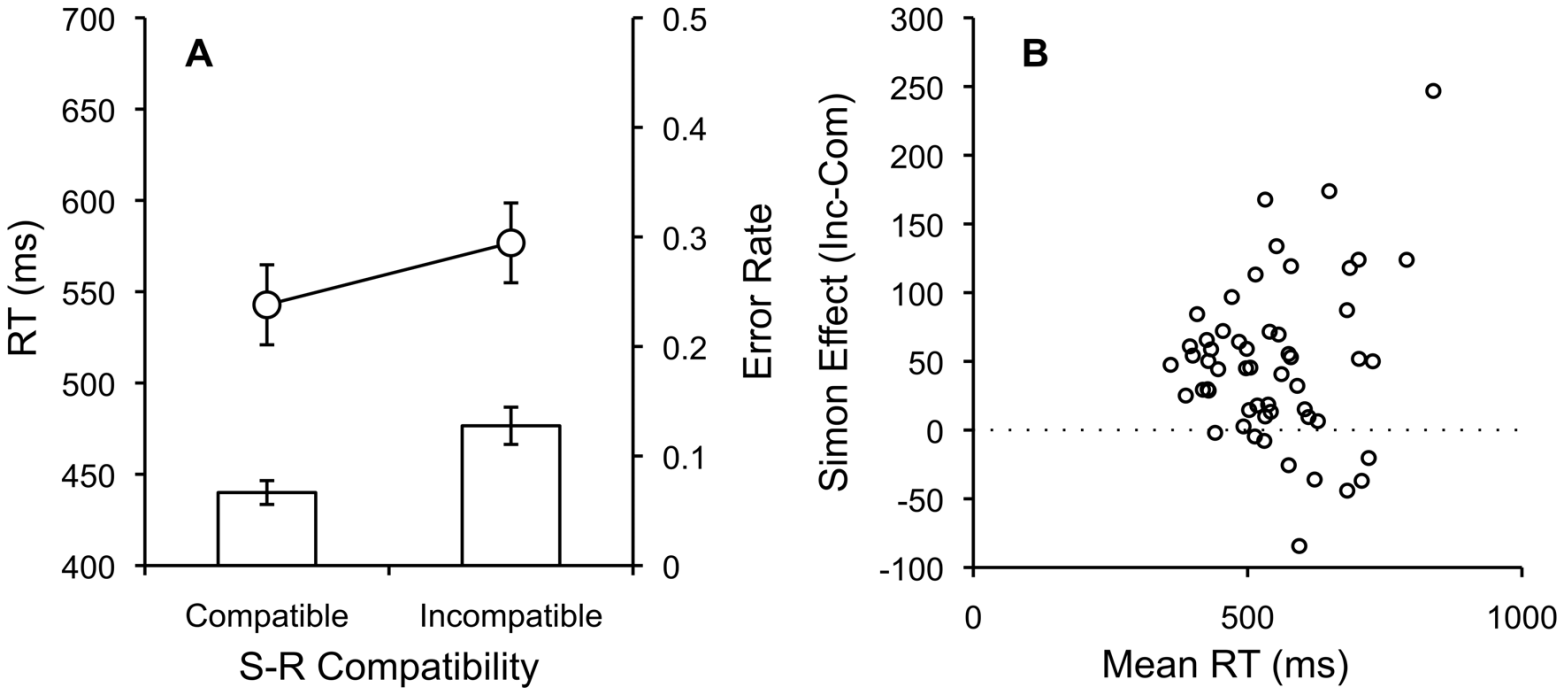
Compatible and Incompatible RTs, Error Rates, and Individual Simon Scores by Mean RT. A. Mean RTs and error rates for compatible and incompatible Simon trials with standard error bars. B. Individual subject Simon scores (incompatible-compatible) plotted as a function of individual subject mean RTs.

The same outlier analyses applied in Experiments 1–3 resulted in removal 3% of the data from each condition. Mean RTs and error rates for each subject in each condition were submitted to separate one-way repeated measures ANOVAs with compatibility (compatible vs. incompatible) as a factor. Figure 4A shows mean RTs and error rates for each condition, and Figure 4B shows individual subject Simon difference scores plotted as a function of mean RT.

RTs were significantly faster for compatible (556 ms) than incompatible (603 ms) trials, F(1,57) = 33.55, MSE = 1851.32, p<.001, η^2^_p_ = .37. Error rates were low overall and significantly lower for compatible (.05) than incompatible (.11) trials, F(1,57) = 36.32, MSE = .0025, p<.001, η^2^_p_ = .39.

The Simon effect was reproduced and was similar to prior laboratory-based reports. For example, in a first training session that had six times as many trials, Proctor & Lu (Exp 1) [31] reported 459 ms for compatible and 481 ms for incompatible trials. Here, the mean RTs are slightly shorter and Simon effect smaller than in the AMT replication, and this difference is likely to due to the limited number of trials involved in the present experiment.

### Section 1: Summary

All of the reaction time tasks chosen for validation purposes were replicated. In addition, error rates were low overall suggesting that participants took the task seriously. When the task required more complex responding, as in the typing version of the Stroop task, RTs were a bit longer than in more simple forced choice tasks. However, this pattern is expected even in the laboratory. In general, all RT patterns appear to be in the expected ranges which have been established in more controlled laboratory settings. Overall, these replications highly recommend AMT as a tool to conduct multi-trial designs that rely on reaction time as a dependent measure.

## Section 2: Rapid Stimulus Presentation

Many attention, perception, and cognition experiments require precise millisecond timing for visual stimulus presentation. Laboratory based research typically employs software and hardware that guarantees precise control over displays. Web browsers equipped with Javascript have the capability to show and hide visual stimuli on the order of milliseconds, however it is unknown whether stimuli are displayed for the exact programmed time values on worker's computers due to the fact that resources are loaded over a Internet connection. Three attention experiments that required relatively short 10–100 ms stimulus presentations were conducted to determine whether standard effects can be replicated using contemporary web-browser technology. These were Posner (or visual) cuing, the attentional blink task, and a subliminal masked priming procedure. For all experiments in this section, participants electronically signed consent forms. The Brooklyn College Institutional Review Board approved the study designs.

## Experiment 5: Visual Cuing & Inhibition of Return

In a visual cuing task, participants are presented with a central fixation cue and asked to detect or identify a target stimulus appearing to the left or right of fixation. Prior to target onset the left or right location is cued by a short and sudden visual percept. The cuing event is often uninformative such that a cue on the left could be followed by a target in either the left or right location with equal probability, and vice versa for cues appearing on the right side. Visual cuing procedures have produced well-established patterns of results reflecting aspect of visual attention [32], [33]. First, when a detection task is used, RTs tend to be much faster (∼300–400 ms) than those observed in the Stroop and Flanker task. Second, when there is short delay between cue and the target (e.g. 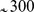 ms) RTs are faster for valid (target appears in cued location) than invalidly cued (target appears in uncued location). Third, when the cue-target interval is longer (e.g. 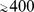 ms) the cuing effect reverses with RTs faster for invalid than valid trials (reflecting inhibition of return for attention [33]). Cuing effects are often relatively small in size. In addition, cue presentation durations can be very short (e.g., 100 ms), and the delay between cue and target must be short enough to measure the positive cuing effect. These task-parameters can easily be programmed in the web-browser based script, but it is unclear whether the intended stimulus presentation times will be error-free on the wide variability of AMT worker computer systems. Replicating visual cuing effects in the context of a simple detection experiment allows us to assess the replicability of studies that depend on precise timing for stimulus presentation.

### Methods

#### Participants

One HIT with 60 assignments was loaded onto AMT. Fifty unique workers completed all 96 trials. Demographic information was not collected and workers remained completely anonymous. Workers were paid $0.10 to complete the task that lasted approximately 5 min.

#### Apparatus, Stimuli & Design

The experiment was presented to workers as an HTML webpage with task flow controlled by JavaScript code running locally in each worker's web browser.

The visual display was composed of three squares arranged horizontally from left-to-right. Each square was 150 px in width and height. Squares were separated by 170 px. Squares were depicted as transparent with a white border, and presented on a black background. The target was a green “X” presented in 40-pt font. When the “X” appeared it was presented centrally inside the left or right square. Cues were white filled squares 100 px in width and height, and appeared centrally inside the left or right squares. The design involved a 2 (validity: validly cued vs. invalid)×4 (Cue-to-target Onset Asynchrony: 100 ms, 400 ms, 800 ms, 1200 ms) factorial design.

#### Procedure

The same screening procedure and webpage used in Experiments 1–4 was employed here.

Each trial began with all three squares presented on screen. The fixation cross appeared in the central square for 500 ms, followed immediately by a cue that was presented in the left or right location. Cue duration was 100 ms. The fixation point remained onscreen during presentation of the cue. After the cue disappeared one of four CTOAs (100 ms, 400 ms, 800 ms, or 1,200 ms) occurred after which the green “X” was presented in the left or right location. Subjects were instructed to detect the “X” by pressing the space bar as quickly and accurately as possible. If subjects' RTs were slower than 500 ms a warning message was displayed that read “respond faster”. The next trial was triggered automatically with a delay of 1,000 ms. Subjects pressed the submit button at the top of the screen after completing the trials.

### Results & Discussion

**Figure 5:**
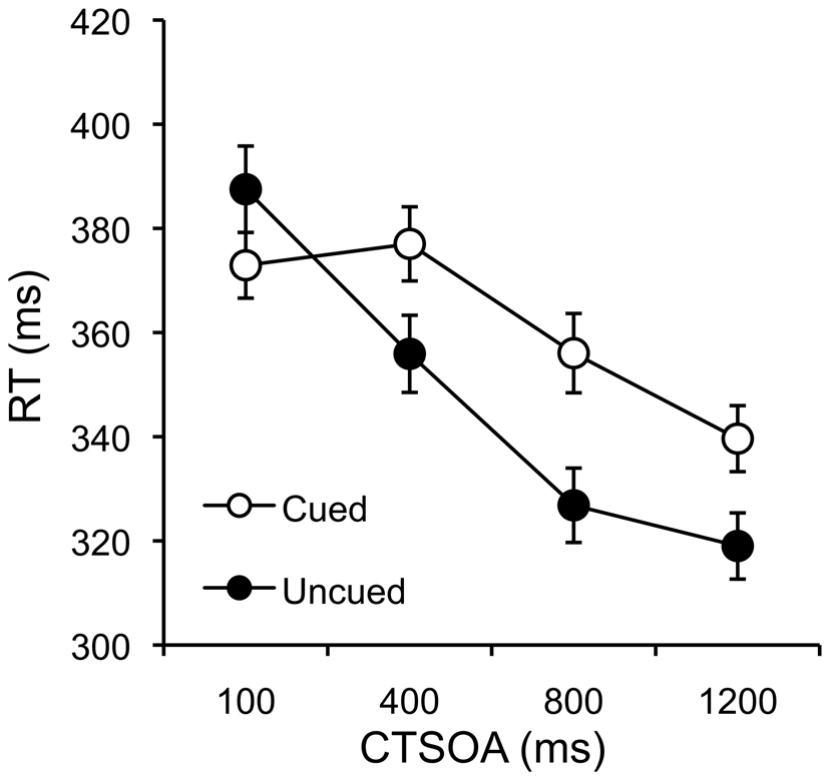
Visual Cuing: Cued and Uncued Mean RTs as a function of CSTOA. Mean RTs for cued and uncued trials as a function of cue-target stimulus onset asynchrony with standard error bars.

The same outlier analysis used in Experiments 1–4 removed 3% of trials from each condition. Mean RTs for each subject in each condition were submitted to a 2 (validity: valid vs. invalid)×4 (CTOA: 100 ms, 400 ms, 800 ms, and 1,200 ms) repeated measures ANOVA. Mean RTs for each condition are displayed in Figure 5.

The main effect of CTOA was significant, F(3,147) = 57.29, MSE = 937.35, p<.001, η^2^_p_ = .54. Mean RTs were 380, 367, 341, and 329 ms across the 100, 400, 800, and 1200 ms delays respectively. This shows expected influences of preparation, with faster RTs for targets appearing with longer cue-target delays. The main effect of validity was significant, F(1,49) = 17.24, MSE = 1148.09, p<.001, η^2^_p_ = .26, but was furthered qualified by the critical validity×CTOA interaction, F(3,147) = 12.95, MSE = 736.63, p<.001, η^2^_p_ = .21. For the 100 ms CTOA condition, validly cued targets were detected faster (373 ms) than invalidly cued targets (387 ms), F(1,49) = 5.07, MSE = 1053.69, p<.028 η^2^_p_ = .09, showing a 15 ms positive cuing effect. For the 400, 800, and 1,200 ms CTOA were obtained for the 100 ms CTOA, and negative cuing effects were obtained for the 400, 800, and 1,200 ms CTOA conditions, validly cued targets were detected slower (358 ms) than invalidly cued targets (334 ms), F(1,49) = 37.34, MSE = 41891.10, p<.001, η^2^_p_ = .43, showing negative cuing effects which are commonly known as inhibition of return. By comparison, the pattern of mean RTs and cuing effects are similar to those reported by Lupiáñez et al. in a laboratory-based study (see their Table 1 [34]).

**Table 1:** The abstract structure of the Shepard, Hovland, and Jenkins (1961) classification problems.

	Classification Category					
Stimulus	I	II	III	IV	V	VI
1 1 1	**A**	**A**	**B**	**B**	**B**	**B**
1 1 2	**A**	**A**	**B**	**B**	**B**	**A**
1 2 1	**A**	**B**	**B**	**B**	**B**	**A**
1 2 2	**A**	**B**	**A**	**A**	**A**	**B**
2 1 1	**B**	**B**	**A**	**B**	**A**	**A**
2 1 2	**B**	**B**	**B**	**A**	**A**	**B**
2 2 1	**B**	**A**	**A**	**A**	**A**	**B**
2 2 2	**B**	**A**	**A**	**A**	**B**	**A**

Each stimulus can be coded as a binary vector along the three stimulus dimensions. The problems differ in how the eight items are assigned to the two categories. The perceptual dimensions (e.g., blue, stripe, border color) were randomly assigned to the abstract stimulus dimensions for each subject.

The fact that visual cuing effects can be replicated using Amazon Turk shows that even small RT effects (∼20 ms) can be reliably measured despite unknown timing variability in stimulus presentation and response recording. Our result suggest that this timing error is small or random and washes out in the averaging over multiple trials and multiple subjects.

## Experiment 6: Attentional Blink

Visual target detection can be impaired for a second target item that appears within 100–500 ms of the first target [35], [36]. This effect is termed the *attentional blink* (AB). Procedures used to measure the AB involve rapid serial visual presentation (RSVP) streams (i.e., sequences of visual images) that require millisecond timing for stimulus presentation control. Typical AB designs involve identifying a target amongst a series of distractors that are presented in a RSVP stream. For example, a stream of 10–15 random letters could be presented with short 100 ms durations for each letter. The first target (T1) letter is a white letter presented amongst black distractor letters. Additionally, a second target (T2), in the form of a black X, is sometimes presented after T1. When T2 is presented its position is varied from immediately after T1 (lag 1), up to any future letter position (lags 2–8). The task involves identifying T1 and then judging whether T2 was presented. The AB is measured for trials where T1 was correctly reported. On these trials, T2 accuracy remains high for lag 1, drops substantively at lag 2, and gradually improves back to ceiling across the remaining lags. The AB effect is commonly thought to reflect attentional processes involved in raising awareness of a stimulus to a conscious level [36].The present experiment replicates a version of the attention blink procedure taken from Klein, Shapiro, & Arnell (Exp 2, [35]).

### Methods

#### Participants

One HIT with 60 assignments was loaded onto AMT. Fifty-two unique workers completed all 96 trials. Demographic information was not collected and workers remained completely anonymous. Workers were paid $0.10 to complete the task which lasted approximately 5 min.

#### Apparatus, Stimuli & Design

The experiment was presented to workers as an HTML webpage with task flow controlled by JavaScript code running locally in each worker's web browser.

The visual display was composed of a grey square 300 px in width and height, placed in the center of the webpage on a black background. Distractor and target letters were presented in the center of the square in 50-pt font. Distractor letters and the second target letter T2 (an X) were always presented in black. The first target letter (T1) was presented in white font.

Letter sequences involved 7–15 pre-target letters and 8 post-target letters. All letters in a stream were unique and randomly ordered from trial-to-trial. The white target (T1) always appeared at the end of the pre-target letter sequence, and the second target (T2, black X) appeared on 50% of the trials in each of the 8 post-target letter positions, with equal proportion. There were a total of 80 trials.

#### Procedure

The same screening procedure and basic webpage used in Experiments 1–5 was employed here.

Each trial began with a fixation cross presented in black in the center of the square for 500 ms. Next the entire stream of letters was presented in series. Each letter was presented for 100 ms and immediately replaced with the following letter. After the stream was completed the square and final letter were immediately blanked. Subjects were cued to identify T1 by pressing the appropriate key on the keyboard. Next, they were instructed to press 1 if the X was present and 0 if the X was absent. The next trial was triggered automatically with a delay of 1,000 ms.

### Results & Discussion

**Figure 6:**
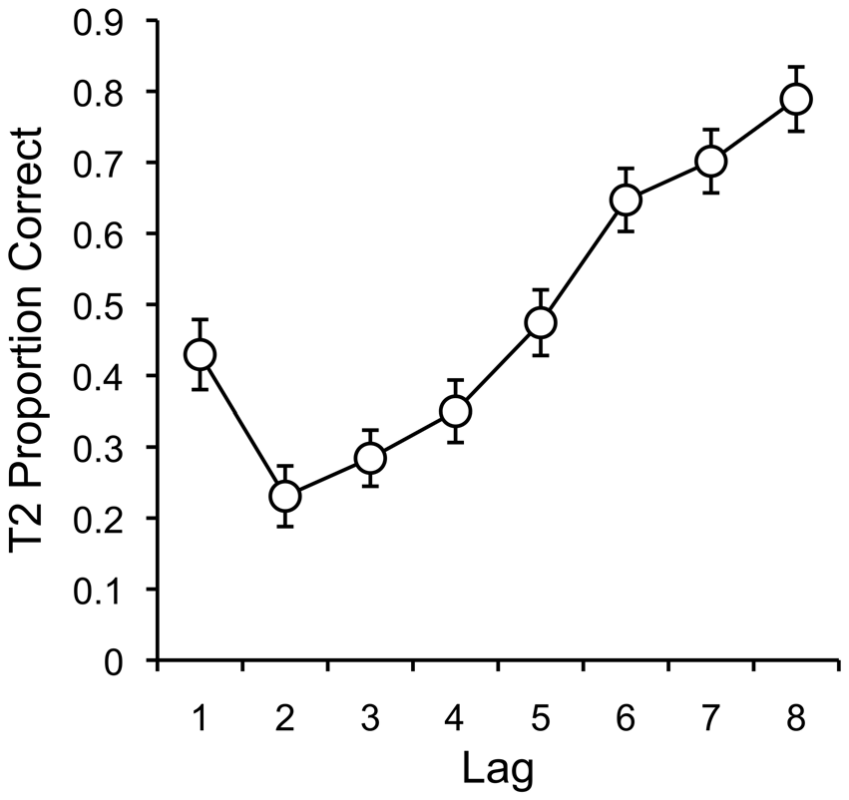
Attentional Blink: Mean T2 Proportion Correct as a function of T1–T2 Lag. Mean T2 (second target) proportion correct as a function of T1–T2 lag with standard error bars.

Only trials in which T1 was correctly identified were considered in the analysis. Mean proportion correct for detecting the second target for in each lag condition was computed for each subject. Means were submitted to a one-way repeated-measures ANOVA with lag as the single factor, and are displayed in Figure 6.

There was a significant effect of lag, F(7,357) = 39.12, MSE = .055, p<.001, η^2^_p_ = .43. The figure shows the characteristic pattern of the AB. Proportion correct was higher for lag 1 (.43) than lag 2 (.23), F(1,51) = 17.04, MSE = .06, p<.001, η^2^_p_ = .25, which is commonly termed lag 1 sparing. Lag 2 shows the worst performance, and proportion correct increases monotonically to lag 8, which shows the highest accuracy (.78).

The hallmark patterns of the AB were replicated. AB experiments use RSVP presentation techniques and require fast stimulus presentation rates. In our experiment, only minimal effort was taken to ensure the timing of stimulus presentation. Nevertheless, it appears that phenomena like the AB may be measured using online using standard web-browser technology with sufficient power to replicate classic laboratory-based findings. This result provides further support to the validity of running experiments online using AMT.

## Experiment 7: Masked Priming

Precise control over stimulus duration is especially important in research involving subliminal perception or visual masking that requires extremely short presentation durations (e.g., on the order of 10 ms). These stimulus durations usually require software and hardware that have been developed and externally tested to ensure correct timing. In the context of the present studies such tests were not conducted, nevertheless it would be interesting to know whether effects that depend on short durations can be replicated in the absence of such rigorous control (with the tradeoff being fast, plentiful participant recruitment). One well-replicated masked priming procedure involves responding to arrow probes (<< or >>) that are primed by briefly presented compatible (e.g., prime: >>; probe >>) or incompatible items (prime: >>; probe <<) [37]. In one study, prime duration was manipulated parametrically involving 16, 32, 48, 64, 80, and 96 ms durations (Exp 2 [38]). The notable finding was that compatibility effects were negative (incompatible RTs faster than compatible RTs) for the 16, 32, and 48 ms durations, but positive (compatible RTs faster than incompatible RTs) for the longer 64, 80, and 96 durations. These results are assumed to reflect qualitative differences in processing of conscious and unconsciously presented stimuli, with response facilitation driven by conscious access to perceptual information and response inhibition driven by automatic self-inhibitory motor control processes. The present experiment attempted to replicate these findings to determine the viability of conducting online research that requires extremely short visual presentations.

### Methods

#### Participants

One HIT with 60 assignments was loaded onto AMT. Thirty-two unique workers completed all 572 trials. Demographic information was not collected and workers remained completely anonymous. Workers were paid $0.50 to complete the task which lasted approximately 15 min.

#### Apparatus, Stimuli & Design

The experiment was presented to workers as an HTML webpage with task flow controlled by JavaScript code running locally in each worker's web browser.

The visual display was composed of a grey square 150 px in width and height, placed in the center of the webpage on a black background. Primes were the stimuli << and >> displayed in black in 20-pt font. The mask was the stimulus ### displayed in black in 20-pt font. Probes were the same as the primes. All stimuli were displayed in the center of the grey square.

There were 48 trials per block and 12 total blocks. Each block employed one six prime durations: 16, 32, 48, 64, 80, and 96 ms. Block order was randomized for each subject.

#### Procedure

The same screening procedure and basic webpage used in the previous experiments was employed here.

Each trial began with a prime presentation followed by a mask for 100 ms. Next, a blank interval was presented for 50 ms. Finally, the probe stimulus appeared for 100 ms and then removed immediately from the screen. Subjects were instructed to press S for the << stimulus and K for the >> stimulus, and to make their responses as quickly and accurately as possible.

### Results & Discussion

**Figure 7:**
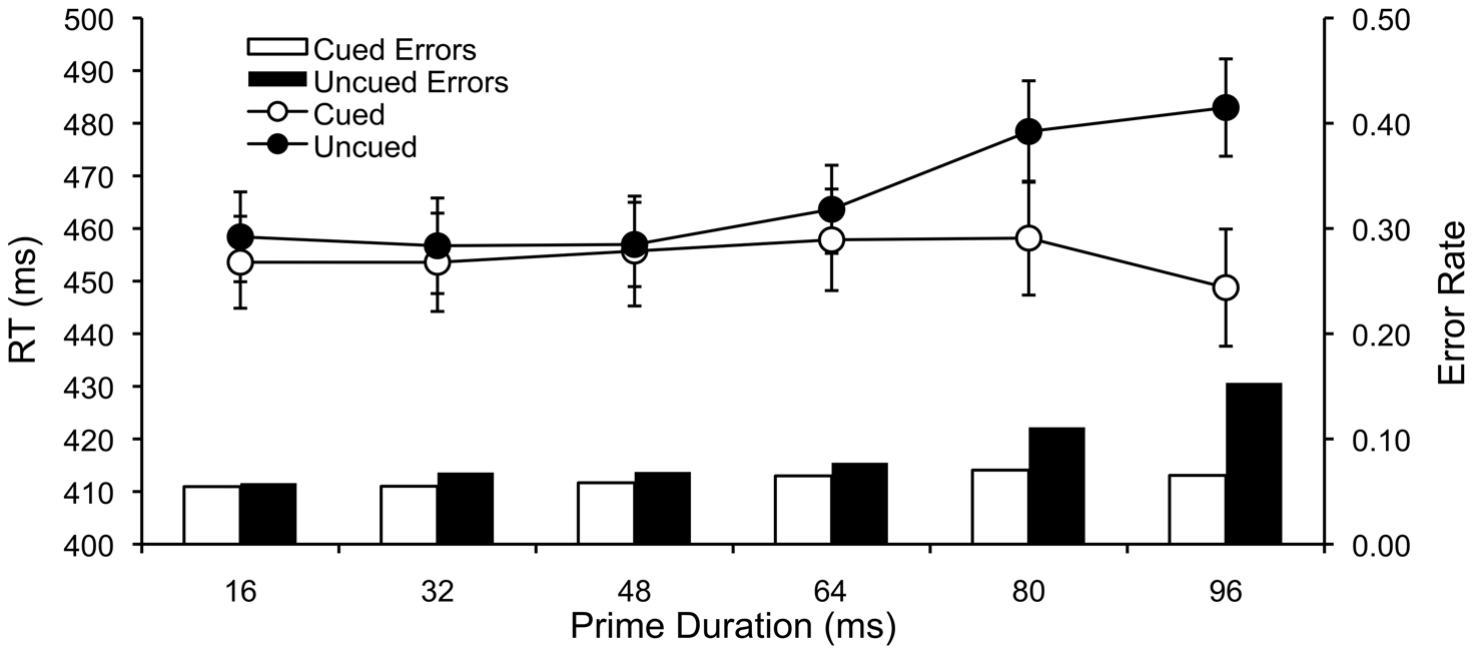
Masked Priming: Compatible and Incompatible Mean RTs and Error Rates Across Prime Durations. Mean RTs and error rates for compatible and incompatible masked prime trials as a function of prime duration with standard error bars.

The same outlier analysis conducted on Experiments 1–6 removed 3% of trials from each condition. Mean RTs for each subject in each condition were submitted to a 2 (compatibility: compatible vs. incompatible)×6 (Prime Duration: 16, 32, 48, 64, 80, & 96 ms) repeated measures ANOVA. Mean RTs for each condition are displayed in Figure 7.

The main effect of compatibility was significant, F(1,32) = 8.04, MSE = 1652.37, p<.01, η^2^_p_ = .20. Compatible RTs (455 ms) were faster than incompatible RTs (466 ms). The main effect of prime duration was significant, F(5,160) = 2.85, MSE = 726.66, p<.017, η^2^_p_ = .08. Mean RTs were 456, 455, 456, 460, 468, and 466 ms, in the 16, 32, 48, 64, 80, and 96 ms prime duration conditions, respectively. The compatibility×prime duration interaction was significant, F(5,160) = 7.10, MSE = 392.48, p<.001, η^2^_p_ = .18. Compatibility effects (i.e., uncued – cued) were not significant for the 16 (5 ms, F(1,32) = 1.73, p<.198, 32 (3 ms, F<1), 48 (1 ms, F<1), or 64 ms (6 ms, F<1) prime duration conditions. However, positive compatibility were significant for the 80 (20 ms, F(1,32) = 7.07, MSE = 957.73, p<.012, η^2^_p_ = .18) and 96 ms (34 ms, F(1,32) = 17.30, MSE = 1116.53, p<.001, η^2^_p_ = .35.) prime duration conditions. A corresponding analysis of error rates was also conducted. The pattern of error rates mimicked the pattern of RTs, but the analysis is not reported for sake of brevity.

The data show a partial replication of Eimer & Schlaghecken (Exp 2 [38]). The original study observed significant negative compatibility effects (i.e., incompatible faster than compatible) for the 16, 32, and 48 ms prime durations. The present replication showed no significant compatibility effects for these prime durations, or for the 64 ms prime duration. As well, the trend in the means was for positive compatibility rather than negative compatibility effects. The original study observed significant positive compatibility effects for the 64, 80, and 96 ms prime duration conditions. The present replication showed significant positive compatibility effects for the 80 and 96 ms prime duration conditions. The fact that compatibility effects were not observed for the 16 to 64 ms prime duration conditions demonstrates important limitations in using web-browser technology to conduct experiments that require fine millisecond control over stimulus presentation. This result is not totally unexpected given the discussion above. Typically, specialized hardware and careful control is needed to ensure stimulus presentation times lower than 50 ms. However, this replication attempt shows the likely limits to behavioral research that can be conducted online using systems such as AMT. Fortunately, only very specific questions regarding visual processing and awareness require this type of stimulus control.

### Section 2: Summary

Experiments 5 through 7 examined experiment designs requiring millisecond control over visual stimulus presentation. Posner cuing effects, attentional blink, and masked priming effects that used relatively long (80 ms or longer) stimulus presentation times were all replicated. Experiment 7 required parametric manipulation of stimulus duration in 16 ms steps, and compatibility effects for prime durations 64 ms and shorter were not observed, likely indicating constraints for conducting experiments that require very short stimulus presentation times. As web-browser technology improves, or if researchers can produce web-based designs that ensure accurate presentation times for very short durations, online subject recruitment may become a valuable resource for such studies in the future.

## Section 3: Learning Studies

The experiments considered so far have involved repeated trials that are independent from one another (i.e., the response on one trial is not related the response to the previous one). In addition, in most cases, the instructions were relatively obvious (e.g., in the Stroop experiment the instructions are simply described by the prompt on every trial). However, other experiments of interest to psychologist require more complex instructions and non-independent trials, as in learning tasks. In this section, we turn our effort to replicating classic learning studies using AMT. Note that in many learning tasks, it is critical that participants understand key concepts (such as the whether the task is changing over time) as well as integrate experience across a series of trials. To foreshadow, our initial investigations into these types of experiments was quite different from the result of the previous section (we failed to replicate in some cases). However, in a series of follow-up studies we explore a number of alternative factors that may have influenced our results.

## Experiment 8: Category Learning

In our first learning experiment with AMT we attempted to replicate Shepard, Hovland, and Jenkins' [39] classic study on concept learning. This is a highly influential experiment that has been replicated many times in different laboratories using slightly different materials [40], [41], [42]. As a summary, Shepard et al. had participants learn one of six different categorical groupings of a set of eight geometric objects. Each of the six groupings varied in difficulty in a way related to the complexity of the criteria needed to correctly partition the items. Interestingly, differences in difficulty among the problems persist despite the fact that, in theory, people could simply memorize the category membership of each of the eight items. This is often taken to imply people are forming more abstract, structured conceptions of the regularity governing the category distinction (e.g., by inferring an explicit rule which determines category membership).

For example, in the first problem (known as the Type I problem) a natural solution is to form a rule along a single stimulus dimension (e.g., “If the object is blue then respond Category A, otherwise respond Category B.”). The Type I problem is usually fairly easy to learn across a sequence of trials, while other problems are more difficult. For example, the Type VI problem is a complicated three-way XOR between the stimulus dimensions and might be best learned by memorizing the category membership of each item. A full description of the abstract structure of the Shepard et al. learning problems is shown in Table 1.

In general, previous research has shown that the Type I problem is reliably learned more easily across trials than is the Type II problem. In turn, Types III, IV, and V are learned more slowly than Type II (within problems III–V, learning rates appear mostly similar). Finally, Type VI is typically the most difficult pattern to learn. The relative rate of learning for these six problems has provided an important constraint on theories of human concept and category learning. For example, most computational models of categorization must account for the relative difficulty of these problems in order to be viewed as a serious theoretical account. In addition, the quantitative (rather than qualitative) shape of the learning curves has been used to test and differentiate models [43], [44]. As a result, this study is a natural candidate for replication using AMT. One practical challenge with conducting this study is that there are six separate experimental conditions and usually each subject should only contribute data to one condition (to avoid possible carry-over effects). In light of this, our goal in Experiment 8 was to see if we could replicate this finding using participants recruited over the Internet.

### Methods

#### Participants

Two hundred and thirty-four anonymous online participants volunteered (N = 38 in each of the six problems), and each received $1.00 via AMT's built-in payment system. In addition, one in ten participants who completed the task were randomly selected for a bonus raffle of $10. This incentive was included to encourage people to finish the task even if they found it difficult, a helpful precaution against people withdrawing from the study in the more challenging problems (e.g., Type VI). An additional 56 participants initiated the experiment electronically, but withdrew before the end for unknown reasons. The data from these participants was not further analyzed. Finally, seven individuals indicated they used pen and paper to solve the task in a post-experiment questionnaire and were excluded (although these participants still received payment). Participants electronically signed consent forms and were debriefed after the experiment. The NYU Institutional Review Board approved the study design.

We conducted our experiment between 1∶30 p.m. EST February 24th, 2012 and 6 p.m. EST February 28th, 2012 (we expect this information may be useful for researchers to know if the demographics of AMT change over time). Data collection was generally paused each evening at around 9 p.m. EST and started again the following morning. A restriction was put in place that participants were located with the United States and had at 95% acceptance rate for HITs. The purpose of this was to increase the probability that the participants were native English speakers who could fully understand the instructions and so we could keep data collection during relatively normal working hours. In addition, our experiment code checked the worker ID and made sure that each unique account could only participate in the task once. People could evade this restriction if they had multiple Amazon accounts, but doing so would be a violation of Amazon's Terms of Use policy.

#### Design

Each participant was randomly assigned to complete one of the six learning problems defined by Shepard et al. [39] and shown in Table 1. The mapping between the stimuli and the abstract structure shown in Table 1 was randomly counterbalanced across participants.

#### Apparatus & Stimuli

The experiment was served to workers as an HTML webpage with task flow controlled by JavaScript code running locally in each worker's web browser. Our software for running AMT experiments is provided at http://github.com/NYUCCL/PsiTurk. Due to an incompatibility with Microsoft Internet Explorer (IE)'s rendering engine, participants using IE were denied access to the experiment and asked to download an alternate (free) browser such as Google Chrome.

The stimuli were simple square objects that varied in the border color (yellow or white), fill color (blue or purple), texture (smooth or rough), and stripe (present or absent). The stimuli we used were developed by Love [45] who normed the constituent dimensions for roughly equal psychological salience using college-aged undergraduates. For each individual, only three of the four dimensions were relevant of the study (the three dimensions in Table 1) and the fourth was held at a fixed value.

#### Procedure

Our replication, although presented in AMT, remained procedurally similar to a highly cited laboratory replication of the Shepard et al. [39] results by Nosofsky et al. [40]. On each trial of the task, one of the eight objects was presented in the middle of the browser window. The participant indicated if the item belonged to category A or B by clicking the appropriate button. Feedback was then presented for 500 ms, which indicated if the response was correct or incorrect.

Trials were organized into blocks of 16 trials. In the rest period between blocks, participants were given information about their performance in the previous block and about how many more blocks remained. The experiment lasted until the participant responded correctly for two blocks in a row (32 trials) or until they completed 15 blocks. Participants were told that the experiment could last as long as 15 blocks, but that they could end early if they correctly learned the grouping quickly. Participants were asked not to use pen and paper.

After completing the task, participants filled out a brief questionnaire that asked if they used any external learning aids (e.g. pencil and paper), if they used any particular strategy, how much they enjoyed the task, and how difficult they thought it was.

### Results & Discussion

**Figure 8:**
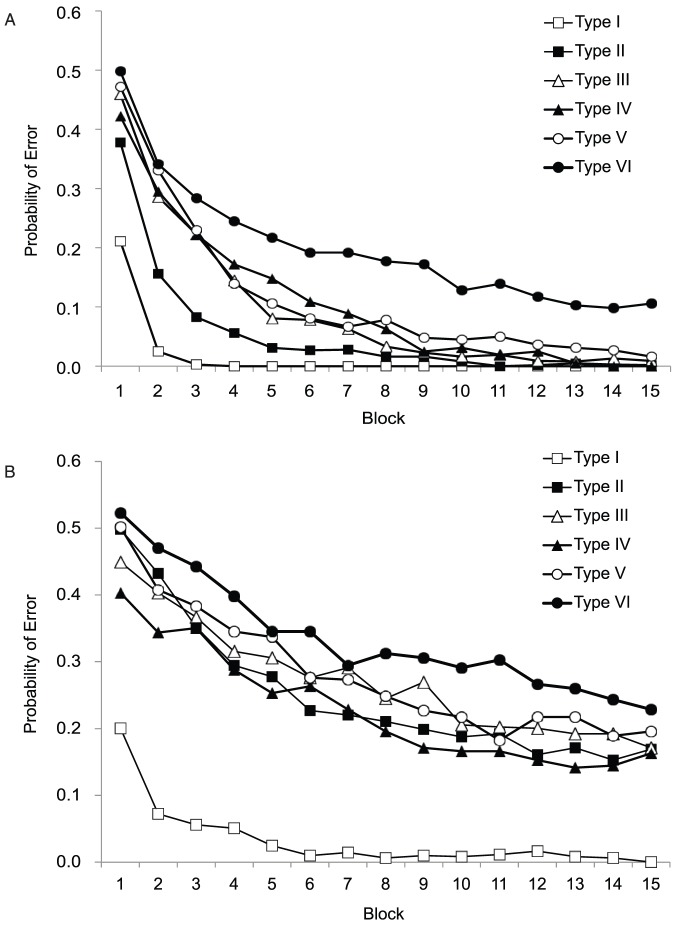
Cognitive Learning: A comparison between the learning curves reported in Nosfosky et al. (1994) data and the AMT replication data in Experiment 8. The probability of classification error as a function of training block. The top panel shows the learning curves estimated by Nosfosky et al. [38] using 120 participants (40 per learning problem) who each performed two randomly selected problems. The right panel shows our AMT data with 228 participants, each who performed only one problem (38 per condition). We ended the experiment after 15 blocks, although Nosofsky et al. stopped after 25. Thus, the Nosofsky et al. data have been truncated to facilitate visual comparison.

Figure 8 shows the probability of making a classification error as a function of training block for each of the six problem types. If a participant reached the performance criterion (one block 100% correct) before the 15th block, we assumed they would continue to respond perfectly for all remaining blocks. Figure 8 is split in two panels. The laboratory data collected by Nosofsky et al. [40] appears in the top panel and our AMT data appear in the bottom panel.

There are several patterns of interest. First, like participants in Nosofsky et al. [40], participants in the AMT experiment learn over trials and reduce the error rate. In addition, the Type I problem was learned very quickly (within the first two or three blocks). In contrast, the error rate for the Type II problem is somewhat higher (and more similar to Types III, IV, and V).

At the same time, in all conditions besides Type I, our participants performed significantly worse than Nosofsky et al.'s [40] participants. For example, in all problems except for Type VI, the probability of error in Nosofsky's study fell below .1 by block 15. In contrast, our error rates asymptote near .2. One hypothesis is that participants on AMT generally learn more slowly, but this would not explain why Type I was learned at a similar rate to Nosofsky (the probability of error drops below .1 by the second block of trials).

**Figure 9:**
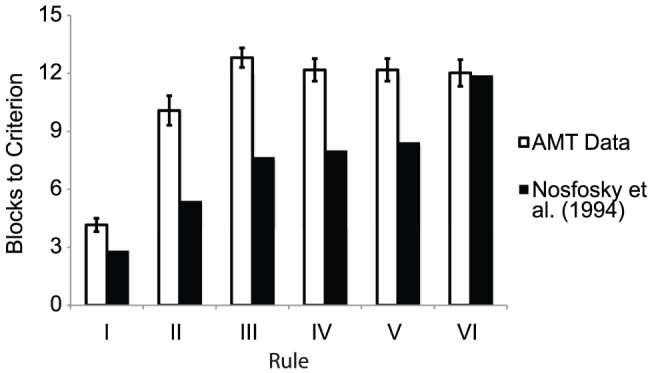
Cognitive Learning: The average number of block to criterion for each problem, an index of problem difficulty. The average number of blocks it took participants to reach criterion (2 blocks of 16 trials in a row with no mistakes) in each problem. The white bars show the estimated average number of blocks to criterion reported by Nosofsky et al. [38].

This rather slower learning rate for the more complex problems is also reflected in Figure 9, which compares the average number of blocks taken to reach criterion both participants in our data and for Nosofsky et al. [40]. In almost every problem, participants on AMT took nearly double the number of blocks compared to Nosofsky et al.'s laboratory study. Closer inspection of the data showed that this was due to a rather large proportion of participants who never mastered the problems at all (taking all 15 blocks). However, this view of the data suggests that Type II was at least marginally easier than Types III–V.

Interestingly, the difficulty of the task (according to Shepard et al. [39] and Nosofsky et al. [40]) did not have a strong impact on people deciding to drop out of the task. To assess this we counted the number of participants who started the experiment but didn't successfully finish as a function of condition. There were four, eight, three, seven, ten, and eight dropouts for problem Types I, II, III, IV, V, and VI, respectively. Thus, the dropout rate does not seem to be systematically related to the problem difficulty (e.g., the smallest number of dropouts was in the Type III problem which, according to the error analyses, was somewhat difficult for participants).

It is also worth noting that we did not attempt any additional post hoc “clean up” of the data (e.g., excluding people who took a long time or who pressed the same key for many trials in a row). While such exclusion may be warranted in certain cases, we didn't have clear a priori hypotheses about which kinds of exclusions would be appropriate for this data. However, given the large percentage of subjects who failed to master the problems within 15 blocks, it is unlikely that there is a simple exclusion criterion that would make our data align well with the Nosofsky et al. [40] replication (without directly excluding people who did not learn).

## Experiment 9: Category Learning and the Effect of Payment Magnitude

The results of Experiment 8 were somewhat mixed. Participants did show learning across trials (e.g., clearly in the Type I problem and as reflected in the overall error rates). However, at least when compared to Nosofsky et al. [40] learning performance in our replication was considerable lower. These results also differ from studies 1–6 which showed relatively similar patterns of online and laboratory data.

One possibility is that if we better incentivized participants' performance, we could get better data. In other words, is the quality of AMT data basically as good as you are willing to pay? As noted by Gosling et al. [3], some AMT workers seem to participate mainly for personal enjoyment, and payment isn't an important issue for these individuals. For example, in their study, they found that a large number of workers would complete a survey for $0.01 (the minimum possible payment).

However, this does not apply universally. Anecdotally, we attempted to run the Shepard et al. [39] study reported above but only offered $0.25 as payment (and no lottery or bonus). In that case we recruited only 1 subject in 12 hours (2 others dropped out after the first block of the task). Thus, workers are influenced to participate by the magnitude of payment and their estimation of the difficulty or length of the task. However, this sensitivity to the possible payment might also influence task performance in theoretically significant ways.

In a second study, we systematically explored how our replication results might depend on how much money the AMT workers are offered. This issue is rarely examined systematically in the laboratory but could have important implications in online data where participants decision to participate may be more strongly influenced by economic concerns (e.g., there are many other tasks available on AMT and the switch costs are low, so the opportunity costs may be more apparent).

Specifically, we repeated the above study with two different incentive structures. We felt our initial payment scheme described above was roughly in line with what we would pay a laboratory subject for a short 15–20 minute task ($1.50 on average). To explore the space of payment options, we created two additional conditions, a low-incentive group that was paid $0.75 and not offered a bonus. A second high-incentive group was offered a guaranteed $2 and a bonus of up to $2.50 based on task performance.

Rather than test all six Shepard et al. [39] problem sets we focused this analysis on the Type II and IV problems, which are often considered to be the two most theoretically significant problems. By comparing the results of this replication with our previous experiment we hoped we could obtain information about the relative effects of payment on the relationship between our online replication and related laboratory studies. In addition, we collected demographic information about participants in this study.

### Methods

#### Participants

Eighty-two anonymous online participants volunteered and were evenly divided between either a low-incentive or high-incentive condition. Within each condition, participants were randomly assigned to either the Type II or Type IV problems (N = 20 or 21 in each condition). In the low-incentive condition each participant received $0.75 via AMT's built-in payment system. There was no bonus or lottery offered for these participants. In the high-incentive condition, participants in were paid a base amount of $2 for completing the experiment and a bonus of up to $2.50. The bonus was calculated as follows: at the end of the experiment, 10 random trials were selected from the participant's data file and each trial where the participant provided a correct response increased the bonus by $0.25. If the participant reached criterion (2 blocks with 100% correct responses) we coded all remaining trials as correct. This placed a relatively stronger financial incentive on quickly mastering the problem compared to either the low-incentive condition or the previous experiment.

An additional twenty participants initiated the experiment electronically, but withdrew before the end for unknown reasons or self-reported using pen and paper to complete the task. As before, a restriction was put in place that participants were located with the United States and had at 95% acceptance rate for previous HITs.

We collected data for the low-incentive condition during a 25 hr period beginning March 9th, 2012 at 5 p.m. EST and ending March 10th at 6 p.m. EST. Data collection was stopped at 9 p.m. EST each evening and began again after 10 a.m. EST. We collected data for the high-incentive condition during a 2 hr period beginning March 20th, 2012 at 3:30 p.m. EST and ending 5:30 p.m. EST.

#### Design

Each participant was randomly assigned to complete one of the six learning problems defined by Shepard et al. [39] and shown in Table 1. The stimuli were simple square objects that varied in the border color (yellow or white), main color (blue or purple), texture (smooth or rough), and stripe (present or absent). As before, the stimuli were developed by Love [45] who normed the constituent dimensions for roughly equal psychological salience. The mapping between the stimuli and the abstract structure shown in Figure 2 was randomly counterbalanced across participants. Only three of the four dimensions were relevant of the study (i.e., the three dimensions in Table 1) and the fourth was held at a fixed value for all eight stimuli.

#### Apparatus & Stimuli

The apparatus and stimuli were identical to Experiment 8.

#### Procedure

The design was mostly identical to the previous study except participants only completed either the Type II or Type IV problem. The procedure was mostly identical to Experiment 8; the only difference was the incentive (high or low).

### Results & Discussion

**Figure 10:**
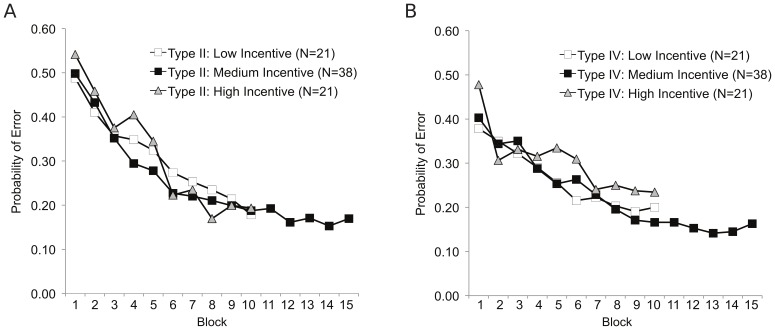
Cognitive Learning: The learning curves for Shepard et al. Type II and IV problems based on task incentives. The probability of classification error as a function of training block, learning problem and incentive for Experiment 9. The incentive structure had little impact on performance within each problem.

Figure 10 compares the learning curves for both the Type II and Type IV problems across three incentive conditions (the medium incentive data are the same as above). The incentive structure of the task had little impact on overall learning rates in the task and does not fundamentally change the impression that the Type II and Type IV problems were learned at a roughly similar rate. There were no significant differences between the incentive conditions in overall error rate for Types II or IV. This result aligns well with Mason and Watts [7], who report that the magnitude of payment does not have a strong effect on the quality of data obtained from online, crowd-sourced systems.

However, the incentive variable did influence the rate of signups (40 subjects were collected in 2 hours in the high incentive condition while it took roughly two days to collect the same amount of data in the low incentive condition). In addition, it strongly influenced the dropout rate. In the high incentive condition, only five participants started the task without finishing (two in Type II and three in Type IV), giving a dropout rate overall of 11%. In contrast, 13 participants in the low incentive condition started but did not finish the task (six in Type II and seven in Type IV), for an overall dropout rate of ∼25%. Again, this result is largely consonant with the conclusions of Mason and Watts [7] in a different task.

## Experiment 10: An Instructional Manipulation Check

Our results so far are interesting, but also suggest caution in using AMT data in cognitive science research. Despite some hints of the classic learning pattern in our data, there were fairly large discrepancies between our study and laboratory collected data. This mostly manifested in significantly worse learning for the more difficult conditions (problems II–VI, relative to the simple one-dimensional rule used in problem I). One concern is that the variable testing environment online contributes to distraction or lack of participant motivation that might negatively impact performance in more challenging cognitive tasks. This would tend to reduce the utility of systems like AMT for research on these topics.

However, rather than give up, we doubled down in our efforts. First, we made some changes to our experiment to be more in line with Nosofsky et al.'s original replication [40]. In particular, we replaced the stimuli developed by Love [44] with the simple geometric figures used by Nosofsky et al. and Shepard et al [39]. Pilot data suggested that the stimulus differences were not the main factor influencing performance but to ensure more comparable results we thought it would be prudent to minimize all differences.

Second, we became concerned that some participants may not have completely understood the instructions; for example, some responses to the post-experiment questionnaire indicated that people believed the rule was changing from one block to the next. It seemed likely that a failure to fully understand the instructions would negatively impact performance, particularly on the more difficult problems.

To address this issue, we incorporated an instructional manipulation check that has been shown to account for unexplained variance in behavioral experiments [46]. This straightforward technique requires the participant to answer non-trivial comprehension questions about the instructions of the experiment before participating. While Oppenheimer et al. [46] introduced somewhat insidious “gotcha” questions into their instructions, we simply presented participants with a questionnaire at the end of the instruction phase which tested knowledge of the basic task and study goals. Correct answers to the questionnaire required a complete comprehension of the goals of the experiment and addressed possible misconceptions (e.g., “Will the rule change on each block?”, “is it possible to get 100% correct?”, “should you use pen and paper to solve the task?”). If a participant incorrectly answered any of the questions, they were asked politely to read the instruction again. This process repeated in a loop until the participant was able to answer all of the comprehension questions correctly.

### Methods

#### Participants

Two hundred anonymous online participants volunteered and were each randomly assigned to a Type I, II, IV, or VI problem (N = 50 in each). Participants were offered $1 to complete the task along with a one in ten chance of winning a $10 bonus (only available if they completed the task). This matches the medium incentive condition used in Experiment 8.

An additional 33 participants initiated the experiment electronically, but withdrew before the end for unknown reasons or self-reported using pen and paper to complete the task. As before, a restriction was put in place that participants were located with the United States and had at 95% acceptance rate for previous HITs. We collected data beginning March 29th, 2012 at 11:30 a.m. EST and ending April 2nd at 5 p.m. EST. Data collection was stopped around 9 p.m. EST each evening and began again after 10 a.m. EST.

#### Apparatus, Stimuli, Design & Procedure

The design was identical to the previous study except participants only completed one of the Type I, II, IV, or VI problems. The only major change was to the stimuli (made to match Nosofsky et al. [40]) and the instructions (detailed above). The procedure was identical to before.

### Results & Discussion

**Figure 11:**
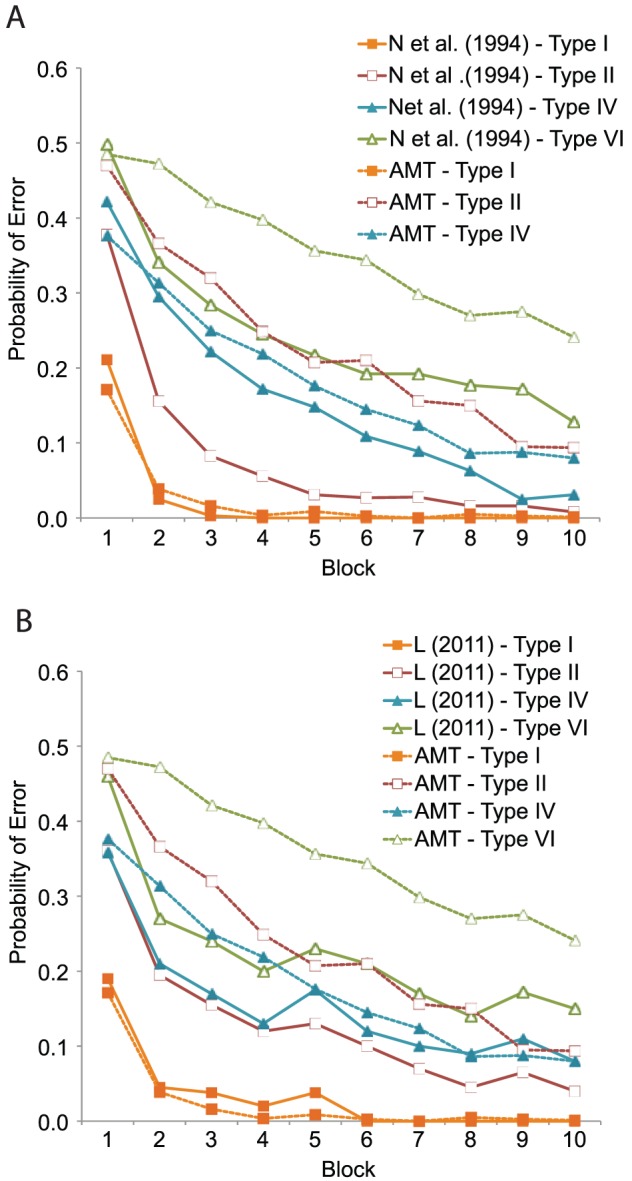
Cognitive Learning: The learning curves for Shepard et al. problems I, II, IV, and VI in Experiment 10. The top panel compared the results of Nosfosky et al. [38] to the results of Experiment 10. The bottom compares the results of Lewandowsky [40] to the results of Experiment 10 giving two different views of the relationship between the online and laboratory based data. Overall, the Type II problem seems more difficult than in previous report (as is the Type VI). However, in general, the instruction manipulation increased the congruence between the online and laboratory data.

Figure 11 (top panel) compares the learning curves for Nosofsky et al. [40] and Experiment 10. The most striking pattern is the closer correspondence between our AMT data and the laboratory-collected data for Types I and IV. These data probably fall within the acceptable margin of error across independent replications of the laboratory study. As an illustration, the bottom panel compares our AMT data to a separate laboratory-based replication by Lewandowsky [42]. Given the intrinsic variability across replications, this suggests the AMT data do a fairly good job of replicating the laboratory-based results. In contrast, the Type VI problem appears more difficult for participants on AMT compared to in the lab. However, at least compared to our results in Experiment 1, the relative ordering of the problems is much more pronounced (i.e., Type I is easier than Type IV which is easier than Type VI).

Despite generally increased alignment between the laboratory data and AMT data, anomalies remain. In particular, the Type II problem seems systematically more difficult for participants in our online sample than in Nosofsky et al.'s [40] laboratory study (e.g., the largest discrepancy between the same colored lines is for the solid red line and dashed red line reflecting the Type II problem).

The finding that Type II is learned roughly at the same rate as Type IV in our online sample is interesting. However, other measures of learning suggested at least a marginal Type II advantage. For example, 100% of participants in the Type I problem reached the learning criterion within the 10 training blocks (2 blocks in a row with 100% correct responses). In comparison, 73.1% reached criterion in the type II problem. However, only 56.4% reach criterion in the Type IV problem and 44.8% reach criterion in the Type VI problem. Interestingly, our finding of similar learning curves for the Type II and IV problems has some precedent in the laboratory literature. For example, as visible in the bottom panel of Figure 11, Lewandowksy [42] found that the Type II problem was learned at roughly the same rate that the Type IV problem. A similar result was reported by Love (2002) [45] who found only a marginal Type II advantage compared to the Type IV problem in a related design. In a series of experiments, Kurtz et al. [47] have argued that the Type II advantage can be explained by the extent to which instructions emphasize verbal rules.

### Section 3: Summary

Overall, our experiments with AMT seem promising, but also raise some interesting issues.

First, it was amazing how much data we could collect in a short period of time. Performing a full-sized replication of the Nosofsky et al. [40] data set in under 96 hours is revolutionary. This alone speaks volumes about the potential of services like AMT for accelerating behavioral research.

Second, it is notable that participants did learn in all conditions (error rate dropped from the beginning to the end of the study in all conditions). This fact was not necessarily a given since people could have chosen to respond randomly. Manual inspection of our data suggests this almost never happened.

Third, many participants were willing to take part in the 15–30 minute study even when offered $0.75 in the low incentive condition. Given that this is about 2–3 times longer than typical HITs on the system suggest there is a reasonable market for recruiting participants. In our high incentive condition, we were able to run as many as 40 participants in 2 hours.

Finally, we replicated the key finding of Shepard et al. [39] and Nosofsky et al. [40] (Type I was easier than Types III–V which are easier than Type VI). Our data were a little less clear than the previously published laboratory collected studies. In general, Type II seemed slightly more difficult than previously reported (at least in our learning curve analysis). We are not sure what to make of this difference, except to point out that a couple recent laboratory studies report a similar pattern [45], [42]. In addition, online participants generally learned more slowly (this was especially true in Experiments 1 and 2 but also showed up in the Type VI condition in Experiment 3). It may be that the slower learning relates to the more diverse participant sample than is typical in laboratory studies (e.g., we did find a slightly negative correlation between performance on the Type II problem and self-reported age).

One of our more practical findings was that building in checks for understanding the instructions is critical for ensuring high quality data. After incorporating those changes, our data began looking more like a publication-quality replication study.

## General Discussion

A quick survey of the cognitive science literature suggests that Internet-based studies have not yet made it fully into mainstream cognitive journals. Based on our findings, we recommend that reviewers and editors should consider accepting behavioral experiments done on AMT as a valid methodology (applying as much scrutiny as they would apply to any behavioral paradigm). Even for extended experiments requiring problem solving and learning, and precise millisecond control for response collection and stimulus presentation, the data seem mostly in line with a laboratory results so long as the experiment methods were solid. At the same time, our cognitive learning experiment raise important concerns about running online studies and our visual priming studies show the limitation in browser-based display technologies.

Despite these concerns, overall, we believe AMT is a revolutionary tool for conducting experiments. It offers the ability to run experiments with large numbers of subjects in a matter of hours. This has the potential to transform behavioral research. Additionally, AMT provides an opportunity to reach a more representative population that varies widely in age, education, and ethnicity and geographic location.

Most importantly, AMT and Internet-based research can lead the way in promoting transparency and reproducibility in cognitive research. Psychologists are under increasing criticism for undisclosed flexibility in data collection and statistical analysis [48]. These concerns are strong enough to have prompted an ongoing, large-scale, open-collaboration effort to replicate the findings from the 2008 issues of Journal of Personality and Social Psychology, Psychological Science, and Journal of Experimental Psychology: Learning, Memory, and Cognition [49]. On this note, it is heartening that the experiments reported here mostly replicated with ease. But still more important is the ease with which it will be possible to replicate new experiments. What Internet-based research lacks in environmental control it makes up in the standardization and control over experiment procedures. Because experiments are program scripts that run on web browsers, access to the code alone is adequate to completely replicate the experiment. This could lead to an era of exhaustive transparency, that is at least if researchers are encouraged and agree to publish their data collection scripts along with their manuscripts. Many journals offer supplemental materials or allow links to supporting online materials and this provides one opportunity to share the scripts used to run their experiments. The code for the reported studies will be made available at the author's websites.

### Suggestions and Advice

To conclude, we would like to offer practical advice based on our experience collecting this data set. On the ethical side, we echo the point made by Mason and Suri [2] that researchers should pay AMT users something close to what is offered to someone to perform the task in the lab. Many companies offer simple HITs on AMT for as little as $.10, but such rates are out of line with what subjects in the lab are offered. While our analysis suggests that lower pay doesn't necessarily affect the quality of the data, we have found that we can recruit participant faster and have fewer dropouts by making the study financially appealing.

Second, experiments that are at least somewhat fun and engaging are likely to be better received. A task involving 5000 discrimination judgments for simple lines or sine-wave gratings will have little appeal to workers, potentially leading to increased dropouts and lower quality of data overall. Studies on AMT compete against all the other interesting things to do on the Internet (e.g., YouTube). We received feedback from many of the participants in Experiments 8–10 who said they found the rule-discovery task to be fun and interesting (although to be fair, others hated it).

We considered various ways to exclude suspicious or odd behavior (e.g., pressing the same key many times in a row or long response times) but ultimately chose not to report these analyses. The problem was that our exclusion criteria were arbitrary. Generally, we do not advocate excluding participants except under the most extremely obvious situations of abuse (e.g., pushing the same button the entire time). As with all empirical studies, restrictions should be decided before data collection and clearly reported in papers to avoid excess experimenter degrees of freedom [48]. Additionally, reporting the time of day and date of data collection may be important as the AMT population may evolve over time.

Most importantly, we found that testing participants' comprehension of the instructions was critical. Prior to including such checks, our data in Experiments 8 and 9 were much noisier. In fact, the instruction check had a considerably more robust effect on the quality of our data than did increasing the payment. There are various means by which experiments can be designed to keep participants abreast of instructions during the task, e.g., by giving accuracy as feedback following each trial, by giving prompts to encourage speeded responding when participants do not meet deadlines, or by giving summary assessments of performance after blocks of trials. Such feedback allows participants to make adjustments to bring performance in-line with intended instructions, and may provide extra motivation to improve performance. In retrospect, these points are intuitive, but they were a lesson worth having sooner rather than later.

Finally, it is important to monitor and record the rate at which people begin an experiment but do not finish. This is typically not a problem in laboratory studies since the social pressure of getting up a walking out of the lab is much higher than it is online. However, dropout rates can interact in complex ways with dependent measures such as accuracy (low performing individuals may be more likely to drop out). We recommend that, perhaps unlike a typical laboratory study, all Internet experiments report dropout rates as a function of condition.

Dropout rate may also depend on task length, financial incentive, and other motivations to complete the task. Our studies validated a range of task lengths from 5–30 min with a range of relatively low financial incentives. Across tasks, dropout rates were not prohibitively high, and we expect that these rates would naturally change to the extent that subjects are given incentive to complete the task at hand. We did not conduct lengthier experiments (e.g., more than one hour long, or multi-day experiments); however, our experience leads us to believe that these types of experiments could be conducted by increasing pay and restricting the experiment to highly motivated and accomplished workers.

In conclusion, AMT is a promising development for experimental cognitive science research. On balance, our investigations suggest that the data quality is reasonably high and compares well to laboratory studies. However, important caveats remain. Hopefully, the quality of the data will continue to remain high as additional researchers start to utilize this resource. If we as scientists respect the participants and contribute to a positive experience on AMT it could turn into an invaluable tool for accelerating empirical research.
